# The Multifarious PGPR *Serratia marcescens* CDP-13 Augments Induced Systemic Resistance and Enhanced Salinity Tolerance of Wheat (*Triticum aestivum* L.)

**DOI:** 10.1371/journal.pone.0155026

**Published:** 2016-06-20

**Authors:** Rajnish Prakash Singh, Prabhat Nath Jha

**Affiliations:** Department of Biological Sciences, Birla Institute of Technology and Science (BITS), Pilani, 333031, Rajasthan, India; University of Minho, PORTUGAL

## Abstract

The present study demonstrates the plant growth promoting (PGP) potential of a bacterial isolate CDP-13 isolated from ‘*Capparis decidua*’ plant, and its ability to protect plants from the deleterious effect of biotic and abiotic stressors. Based on 16S rRNA gene sequence analysis, the isolate was identified as *Serratia marcescens*. Among the PGP traits, the isolate was found to be positive for ACC deaminase activity, phosphate solubilization, production of siderophore, indole acetic acid production, nitrogen fixation, and ammonia production. CDP-13 showed growth at an increased salt (NaCl) concentration of up to 6%, indicating its potential to survive and associate with plants growing in saline soil. The inoculation of *S*. *marcescens* enhanced the growth of wheat plant under salinity stress (150–200 mM). It significantly reduced inhibition of plant growth (15 to 85%) caused by salt stressors. Application of CDP-13 also modulated concentration (20 to 75%) of different osmoprotectants (proline, malondialdehyde, total soluble sugar, total protein content, and indole acetic acid) in plants suggesting its role in enabling plants to tolerate salt stressors. In addition, bacterial inoculation also reduced the disease severity caused by fungal infection, which illustrated its ability to confer induced systemic resistance (ISR) in host plants. Treatment of wheat plants with the test organism caused alteration in anti-oxidative enzymes activities (Superoxide dismutase, Catalase, and Peroxidase) under various salinity levels, and therefore minimizes the salinity-induced oxidative damages to the plants. Colonization efficiency of strain CDP-13 was confirmed by CFU count, epi-fluorescence microscopy, and ERIC-PCR-based DNA fingerprinting approach. Hence, the study indicates that bacterium CDP-13 enhances plant growth, and has potential for the amelioration of salinity stress in wheat plants. Likewise, the results also provide insights into biotechnological approaches to using PGPR as an alternative to chemicals and pesticides.

## Introduction

Salinity is the most common abiotic stressor, which severely affects the plant growth and productivity. Presently, more than 6% of the total land area of the world is salt affected especially in arid and semiarid zones [1]. Salinity affects 19.5% of irrigated land and 2.1% of dry land agriculture worldwide. In India, salinity alone affects 7 million hectares of land [2]. Accumulation of salt-rich water decreases the osmotic potential, thereby decreasing the availability of water to the plants. In addition to deleterious osmotic effects, salt stress also causes the toxic ionic effect to the plants. Plants subjected to salinity stress generate the reactive oxygen species (ROS) causing oxidative damages that adversely affect the plant development [3].

Several approaches are in progress to ameliorate the salinity stress with special attention to the use of plant growth promoting rhizobacteria (PGPR) [4]. Term PGPR was first introduced in 1978 by Kloepper and colleagues to a group of beneficial bacteria that promotes plant growth by enhancing the acquisition of nutrients, production of phytohormones, phosphate solubilization, nitrogen fixation, and siderophore production. Microorganisms with such beneficial features enhance seed germination and promote plant growth [5]. Microbe-mediated phosphate solubilisation and ammonia production directly support plant growth as they act as macronutrient, whereas phytohormone like indole acetic acid (IAA) accelerates root growth, and thus enhances uptake of nutrients [6]. Bacterial siderophore, chitinase, and HCN produced in the rhizosphere can indirectly support plant growth by suppressing hazardous effects of biotic stressors.

PGPR are soil-inhabiting non-pathogenic organisms, which not only promote the plant growth but also trigger systemic resistance, the so-called induced systemic resistance (ISR), against various phytopathogens. Hence, use of these beneficial bacteria can be used as an alternative to chemical fertilizers, pesticides and other agricultural supplements [7]. In addition, PGPR with ACC deaminase activity have an additional benefit by which it reduces the level of stress-induced ethylene in host plants, and stimulate plant growth [8]. Experimental evidence suggests that these bacteria promote plant growth in terms of osmotic balance, altering root size and morphology, and increased uptake of nutrients adjusting the nitrogen metabolism [9]. The effectiveness of ACC deaminase enzyme had been reported in ameliorating various environmental stressors like flooding, heat, drought, metal contamination, organic pollutants, wounding, pathogen and insect infection [10,11]. Different bacterial genera possess a different level of enzyme activity under various environmental conditions. However, organisms with ACC deaminase activity approximately >20 nmol α-ketobutyrate (KB) mg^-1^ h^-1^ is sufficient to alleviate growth inhibitory effects of stressors [12]. For instance, *Pseudomonas fluorescens* strain TDK1 with ACCD activity enhanced salt resistance in groundnut plants as compared to other *Pseudomonas* strains lacking ACC deaminase activity [13].

The increase in soil salinity also induces the cellular generation of reactive oxygen species (ROS) such as superoxide (O_2_^-^), hydroxyl radicals (OH^•^), and hydrogen peroxide (H_2_O_2_), which cause membrane degradation, lipid peroxidation and several other physiological and biochemical alteration leading to cell death [14]. To cope with salinity-induced oxidative stress, plants develop several defense mechanisms. Superoxide dismutase (SOD), catalase (CAT), and peroxidase (POX) are the important antioxidant enzymes which detoxify ROS-induced cellular damages and protect crop plants under oxidative stress conditions [15]. A significant increase in the activities of antioxidant enzymes following inoculation with diazotrophic bacteria such as *Azospirillum* and *Azotobacter* has been reported [16]. However, the exact role of microorganisms to augment the anti-oxidative enzyme machinery is at a preliminary level.

Plants growing under salt stress have developed complex physiological and biochemical mechanisms to accumulate the compatible solutes (osmolytes) for maintaining the intracellular osmotic homeostasis [17,18]. The accumulations of these osmolytes maintain turgor pressure and stabilize the various macromolecular structures against the salinity-induced physiological drought stress. Proline, an important osmolyte, contributes to osmotic adjustment by mitigating the ROS-induced oxidative damage [17]. The increase in proline maintains the cellular osmolarity that provides necessary turgidity for cell expansion under stress conditions [19]. Inoculation of PGPR strains *Pseudomonas pseudoalcaligenes*, and *Bacillus pumilus* increased the proline content in rice variety GJ-17 under different level of salinity as compared to uninoculated plants [20]. Besides proline, the accumulation of soluble sugars also maintains the intracellular turgidity [21].

*Capparis decidua* (Forsk.) Edgew. commonly known as Kair, an important shrub having fair tolerance to alkalinity and salinity, is widely distributed in arid and semi-arid tracts in India. The plant also possesses the unique capacity to tolerate drought and heat stress, and thus plays an important role in the rural economy of places like Rajasthan, India [22]. Wheat is an important staple crop of India and grown widely grown across the world. Salinity stress leads to a major drop in wheat grain yield ranging from 20 to 43% with an overall average loss of 40%. Therefore, the present study was designed to screen PGPR associated with *Capparis decidua*, and to characterize its/their plant growth promoting (PGP) abilities in wheat (*Triticum aestivum*) under saline stress conditions. The objective of the present study was to analyze the possible role of the bacterial isolate in conferring resistance to salinity stress in wheat plants. To evaluate the possible roles of the antioxidant defence system and osmolytes for salinity tolerance, we investigated the activities of several antioxidant enzymes (SOD, POX, and CAT) as well as accumulation of major solutes, including proline (Pro), total soluble sugar (TSS) and total soluble protein (TSP) following inoculation of selected bacterium.

## Materials and Methods

### Isolation of ACC deaminase producing rhizospheric bacteria

The ACC deaminase producing rhizospheric bacteria were isolated from *Capparis decidua* (Kair) commonly growing in the desert of Rajasthan (28.13°N, 75.4°E). Rhizospheric sample was collected from abandoned place in Rajasthan, India, for which no permission was required. Specific government policy defining the use of commonly and naturally grown herb plant is not available or lacking in India. We confirm that the field studies did not involve endangered or protected species of plants. For the isolation, ten gram (g) of the rhizospheric soil was collected by uprooting the plant roots, and the soil attached to roots were scraped using forceps. Finally, 1 g of the soil was mixed with 50 ml of sterilized PAF medium (Composition: per litre, 10 g; proteose peptone, 10 g; casein hydrolysate, 1.5 g; anhydrous MgSO_4_, 1.5 g; K_2_HPO_4,_ and 10 ml; glycerol), and incubated on a shaker at 200 rpm set at 30°C. Following incubation, 1 ml aliquot of this culture was transferred to 50 ml sterile DF (Dworkin and Foster) minimal salt medium (Composition: per litre, 4.0 g; KH_2_PO_4_, 6.0 g; Na_2_HPO_4_, 0.2 g; MgSO_4_.7H_2_O, 2.0 g; glucose, 2.0 g; gluconic acid, 2.0 g; citric acid, Trace elements: 1 mg; FeSO_4_.7H_2_O, 10 μg; H_3_BO_3_, 11.19 μg; MnSO_4_.H_2_O, 124.6 μg; ZnSO_4_.7H_2_O, 78.22 μg; CuSO_4_.5H_2_O, 10 μg; MoO_3_, pH 7.2), serially diluted to 10^−6^, and incubated at 30°C for 24 h [23]. Dilution of the culture was plated on sterile DF salt minimal medium supplemented with 3.0 mM ACC (Sigma-Aldrich, India) as the nitrogen source. Morphologically different bacterial colonies were further quantified for ACC deaminase activity. Based on luxuriant growth on selective medium, isolate CDP-13 was selected for further study and subcultured for several generations on DF-ACC agar plate to ensure its ability to use ACC as carbon and nitrogen source. The isolate CDP-13 was assayed for ACC deaminase activity and other plant growth promoting properties as described below. Glycerol stock (15% w/v) of the isolate was prepared and stored at -70°C until further use.

### ACC deaminase activity

ACC deaminase production was assayed following the standard protocol [12]. ACC deaminase activity of the cell-free extract was estimated by monitoring the amount of α-ketobutyrate (α-KB) generated by enzymatic hydrolysis of ACC. A stock solution of ACC (0.5 M) was prepared in sterile distilled water and filtered through a 0.25 μm pore size membrane, and stored in sterile tubes at -20°C. Bacterial isolate was cultured in DF-ACC (3 mM) medium and incubated at 30°C for 72 h. After centrifugation, the retrieved cell pellets were subsequently washed with 0.1 M Tris-HCl (pH 7.6) and 0.1 M Tris-HCl (pH 8.5) respectively. Finally, 30 μl of toluene was added and vigorously vortexed for 30 s. Two hundred μl of toluenized cells were mixed with 20 μl of 0.5 M ACC and incubated at 30°C for 15 min. Following addition of 1 ml of 0.56 M HCl, the mixture was again vortexed and centrifuged for 5 min at 10,000g at room temperature. One ml of the supernatant was mixed with 800 μl of 0.56 M HCl and 300 μl of 2, 4 di-nitrophenylhydrazine and incubated at 30°C for 30 min. Following addition of 2 ml of 2 N NaOH, the absorbance of the mixture was measured at 540 nm in a UV-Vis spectrophotometer (Jasco Corporation, Japan). The measured value was compared with a standard curve of α-ketobutyrate (Sigma-Aldrich, USA) ranging between 0.1 and 1.0 μmol.

### Screening for other PGP traits

Phosphate solubilization assay of the bacterium was determined in National Botanical Research Institute’s Phosphate Medium (NBRIP) containing insoluble tricalcium phosphate [24]. For quantification, isolates were grown in liquid NBRIP broth medium and incubated at 30°C±2 for 5 days. The solubilized phosphate was quantified as per the standard protocol [24]. The IAA production of the bacterium was estimated using Salkowsky’s reagent [25]. Estimation of gibberellic acid was done by spectrophotometric method [26]. For siderophore assay, test organism was spot inoculated on chrome azurole S (CAS) agar plate and incubated for 4–5 days at 30°C. The appearance of the yellowish or orange zone around bacterial growth was considered positive for siderophore production [27]. Ability to fix atmospheric nitrogen was evaluated by growing on N free JNFb^-^ medium following the standard protocol [28]. Screening for ammonia production was performed by Nessler’s reagent method [29]. For HCN production, the isolate was streaked onto tryptic soy agar plate containing an alkaline picric solution (2.5 g l^-1^ picric acid, 12.5 g l^-1^Na_2_CO_3_, pH 13.0). The plates were sealed with parafilm and incubated at 28°C±2 for 4 days and observed for color change [30].

### Genetic characterization and phylogenetic analysis

Genomic DNA of CDP-13 was isolated by using Qiagen DNA extraction kit (Qiagen, USA). For identification, 16S rRNA gene was amplified in a polymerase chain reaction with universal primers 27F1 (5′-AGAGTTTGATCMTGGCTCAG-3′) and 1494Rc (5′-TACGGCTACCTTGTTACGAC-3′) using genomic DNA as template. The PCR mixture (25 μl) contained 3 μl templates, 5 μl of 10 X Taq DNA polymerase buffer, 3 μl of 2.5 mM dNTP, 0.5 μl of 3U Taq DNA polymerase, 20 pmol of forward & reverse primers. Primers were synthesized at Eurofin Genomics Labs Ltd (Eurofin, India). The PCR was performed in a DNA thermal cycler (T100 BIO-RAD, USA) with initial denaturation for 3 min at 94°C, 30 cycles each consisting of denaturation for 1 min at 94°C, primer annealing for 1 min at 54°C, extension at 72°C for 5 min, and a final elongation of 5 min at 72°C. An aliquot of 3–4 μl of PCR reaction product was electrophoresed on a 1% agarose gel containing ethidium bromide (10 mg/ml in H_2_O), and the DNA band was visualized under the UV light in a gel documentation system (BioRad, India). The resulting 1.5 Kb amplicon was purified using a DNA purification kit (Quiagen, USA) and sequenced at Xcelris Genomics Labs Ltd (Xcelris, India). The nucleotide sequences were compared against GenBank database using the NCBI BLAST algorithm and deposited in the NCBI database (http://www.ncbi.nlm.nih.gov/BLAST). Taxonomic affiliation of the isolate was assigned using RDP database (http://rdp.cme.msu.edu/seqmatch/seqmatch_intro.jsp) at 98% threshold of 16S rRNA gene sequence. A phylogenetic tree was constructed using Software MEGA 6.0 and aligned using CLUSTAL-X. The pairwise evolutionary distance was constructed by the Neighbor-Joining method with the bootstrap of 500 replicates to cluster the associated taxa.

### Biochemical Characterization

Isolate was screened for various biochemical tests such as Gram staining, starch agar test, and IMViC (Indole, Methyl-Red, Voges-Proskauer, citrate utilization) tests. Activities for some enzymes such as catalase, oxidase, lipase, amylase, protease, and nitrate reductase were tested as per standard protocols [31]. Screening for sensitivity to various antibiotics namely gentamicin, kanamycin, tetracycline, vancomycin, and chloramphenicol was tested by the Antibiotic sensitivity kit (HTM 002, Himedia). Similarly, carbohydrate utilization efficacy of the isolate was performed by Himedia carbohydrate utilization kit (KB 009, Himedia). For chitinase assay, the isolate was spot inoculated on chitin agar plate amended with 2% phenol red and incubated for 120 h at 28°C±2 [32]. The presence of different types of motility was tested using the standard method described earlier [33]. The isolate CDP-13 was evaluated for its ability to tolerate salt stress by growing it in DF medium amended with different concentrations of NaCl (0.5% to 10%) at 30°C for 5 days. Similarly, the isolate was incubated at different temperatures in the range of 30°C to 50°C. The growth of the bacterium was measured at every 24 h by taking absorbance of the culture at 600 nm in a UV-Vis spectrophotometer in triplicate sets.

### Test of antagonistic activity: dual culture assay

The antagonistic activity of CDP-13 was evaluated based on the inhibition of fungal mycelium in the dual culture assay [34]. A piece of mycelium (5mm) of *Fusarium graminearum* and *Fusarium monaliformiae* was placed on one side of a Potato dextrose agar (PDA, Himedia, India) plate, and the test isolate (CDP-13) was streaked on the other side of medium. The PDA plate was incubated at 28°C for 7–10 days. The antagonistic effect of the bacterial isolate was confirmed by a zone of inhibition formed between the isolate and fungal strains used in the test. The dual culture was performed in three replicates.

### ISR screening on wheat plants (Water-agar assay)

Wheat seeds were surface sterilized with 70% ethanol for 1 min followed by treatment with 1% sodium hypochlorite for 10 min. Seeds were washed three times with distilled water to remove any traces of bleach solution. CDP-13 was grown in Luria-broth medium (Himedia, India), and the bacterial culture was adjusted to the optical density of 0.15 at 600 nm for further application. Seeds treated with sterile distilled water were used as a control. Seeds were placed in plant tissue culture dishes (Tarson, India) containing water agar medium and incubated in a growth chamber at 25°C under a 16 h light /8 h dark cycle. After 6 days of seed germination, a mycelium piece of *F*. *graminearum* or *F*. *moniliforme* was placed in the center of seedlings. Each culture dish was placed on 10 seedlings with three set of replications. The degree of disease symptom of wheat plants was determined by visual inspection 2 weeks after inoculation [34]. Disease severity was determined on the following scale; 0 = no symptom, 1 = less than10% of leaf area with lesions, 2 = 10–25% of leaf area with lesions, 3 = 25–50% of leaf area with lesions, 4 = 50–75% of leaf area with lesions, and 5 = 75% or more severe lesion or dead leaves.

### ISR screening (pot study)

Surface sterilized wheat seeds were grown in the pots containing 300 g of autoclaved soil in the controlled environment of plant growth chamber. One week after the germination, a mycelial piece of fungi *F*. *graminearum* or *F*. *moniliforme* was planted in the soil around the roots of germinated seedlings. The degree of disease symptoms was determined by visual inspections as described above. The data of plant growth disease severity were collected after two weeks of infection. The data was recorded as per mentioned scale described in above section.

### Effect of CDP-13 on growth of wheat plant

Evaluation of plant growth experiment was conducted in pot study under the effect of NaCl and bacterium inoculation. The soil was sterilized by autoclaving at 121°C for 1 h for three consecutive days to kill the entire microorganism and their spores, and 400 g of sterilized soil were filled in plastic pots. Wheat seeds (*Triticum aestivum* L) were surface sterilized by soaking in 1% Sodium hypochlorite (NaOCl) solution for 3 min and 70% ethanol for 2 min followed by three times washing with sterilized water. For inoculum preparation, the bacterial isolate was grown in Luria-broth for 24 h at 30°C. The obtained biomass was washed with PBS buffer for three times and finally adjusted with sterile 0.03 M MgSO_4_ to the absorbance of 0.15 at 600 nm. Sterile glass petri-dishes containing 15 seeds were incubated at room temperature with following treatment: Sterile 0.03 M MgSO_4_ as control and bacterial suspension of 0.15 OD (optical density).as a bacterial treatment. Plants were grown in a growth chamber at 16:8 h light-dark period with 140 μmol m^−2^s^−1^ of light supplied for 15 days after germination. Hoagland media was used for imposing the salt stress as well as providing the nutrient solution. Pots were arranged in completely randomized block design way. After the study time, growth parameters of the plant such as shoot length, root length, fresh weight, and dry weight were recorded for each replicate. To determine the dry weight, shoots and roots were oven-dried separately at 60°C for 48 h. For chlorophyll estimations, fresh leaf samples of 0.5 g were homogenized in 10 ml of 80% acetone and pigments were extracted and quantified [35]. The absorbance at 645 and 663 nm was measured on a UV–Vis spectrophotometer (Jasco Corporation, Japan) and concentrations were expressed as mg g^-1^ fresh weight using following formula.

$$\text{Chl}a=12.7\;A_{663}-2.59\;A_{645}$$

$$\text{Chl}b=22.9\;A_{645}-4.67\;A_{663}$$

### Biochemical analysis

#### Proline estimation

Proline content in the leaves was determined by following the standard protocol with minor modifications [36]. Fresh leaves of 0.5 g were homogenized in 3 ml of 5% (w/v) sulfosalicylic acid and centrifuged at 8,500g for 10 min. Supernatant of 500 μl was made up to 1 ml with sterile water and gently vortexed with 2 volume of 2% ninhydrin. The mixture was boiled for 30 min at 100°C. After cooling, an equal volume of toluene was added to the mixture and upper aqueous phase was used for taking absorbance at 520 nm in a spectrophotometer (Jasco Corporation, Japan). The proline content was calculated by comparing with a standard curve using L-proline as standard (Sigma -Aldrich USA).

#### Lipid peroxidation

Lipid peroxidation was determined by estimating the malondialdehyde (MDA) content produced by thiobarbituric acid reaction with minor modification [37]. Briefly, 1 ml of the alcoholic extract prepared with 0.5 g of leaves was mixed with 1 ml of 0.5% thiobarbituric acid containing 20% trichloroacetic acid. The mixture was heated up to 90°C for 30 min in a water bath. After cooling at room temperature, sample was centrifuged at 5,000 g for 5 min and absorbance was measured at three wavelengths i.e. 400, 532 and 600 nm. After subtracting the non-specific absorbance, the MDA concentration was determined by its molar extinction coefficient (155nm^-1^cm^-1^) and the results were expressed as mmol MDA g^-1^ FW.

#### Total soluble sugar

Total soluble sugar was estimated by anthrone reagent [38]. Alcoholic leaf extract (0.1 ml) prepared by homogenizing 0.5 g leaf with 3 ml of 80% ethanol was mixed with 3 ml of freshly prepared anthrone reagent and placed in a boiling water bath for 10 min. The absorbance of the resultant sample was measured at 620 nm for which 20–400 μg ml^-1^ of glucose was used as a standard for making calibration curve for quantification of soluble sugar synthesized in plants.

#### Auxin and protein content

Similarly, an alcoholic extract of shoot tissue was used for measuring the auxin content [39].One ml of alcoholic extract was mixed with 2 ml of Salkowsky reagent in the dark and incubated for 20 min. The absorbance was measured at 535 nm and compared with the standard curve of IAA. For the estimation of total protein content, plant tissue of 0.5 g was homogenized in extraction buffer containing [50 mM Tris-HCl (pH 8.3), 1 mM EDTA, 3 mM DTT, 0.08% ascorbic acid, 1 mM PMSF]. The total protein was quantified by Bradford method [40].

### Measurement of salinity induced oxidative damage

#### Detection of hydrogen peroxide (H_2_O_2_)

The level of H_2_O_2_ levels in both control and bacterium-treated plants were estimated [41]. A 0.2 g leaf tissue was extracted with 5 ml of 50 mm phosphate buffer (pH 6.5) and centrifuged at 7,000g for 20 min. The extracted solution (3 ml) was then mixed with 1 ml of 0.1% titanium sulfate in 20% H_2_SO_4_ and again centrifuged at 7,000g for 15 min. The intensity of the yellow colored solution was measured at 410 nm. An extinction coefficient of 0.28 μM^-1^ cm^-1^ was used to calculate the extent of H_2_O_2_ and expressed as μmol g^-1^ fresh weight.

#### Estimation of Membrane Stability Index (MSI)

Membrane stability index was estimated by taking 0.2 g leaf tissue in 10 cm^3^ of DDW in two sets. One set was heated at 40°C for 30 min in a water bath, and the electrical conductivity bridge (C1) was measured on a conductivity meter. The second set was boiled at 100°C in a boiling water bath for 10 min, and its conductivity was also measured on the conductivity bridge (C2). MSI was calculated using the formula:

MSI=[1−(C1C2)]×100

#### Determination of the formation rate of O_2_^-^

To measure the O_2_^-^ content, the control and bacterium treated leaves (0.5 g) were ground in liquid nitrogen. The obtained powder was suspended in phosphate-buffered saline (PBS) buffer (50 mM, pH 7.8). After centrifugation at 12,000g for 15 min, the supernatant was used for O_2_^-^ content measurements.

### Antioxidant enzyme activities

The activity of SOD was determined in control and bacterium-inoculated plants by the photoreduction of nitroblue tetrazolium (NBT). The reaction mixture contained 50 mM phosphate buffer (pH 7.8), 0.1 mM EDTA, 13 mM methionine, 75 mM NBT, 2 mM riboflavin and 100 ml of enzyme extract. Riboflavin was added as the last component, and the reaction was started by placing the tubes under two 15 W fluorescent lamps. The reaction was terminated after 10 min by removing the reaction tubes from the light source. Reaction products were measured at 560 nm. The volume of the supernatant corresponding to 50% inhibition of the reaction was assigned a value of one enzyme unit and activity was expressed as unit mg^-1^ protein.

For the catalase activity, leaf material was homogenized with 50 mM phosphate buffer (pH 7.0) and the homogenate was centrifuged at 8,000g for 20 min at 4°C. Twenty ml of enzyme extract was added to 3 ml of hydrogen peroxide phosphate buffer (pH 7.0). The time required for the decrease in the absorbance at 240 nm from 0.45 to 0.40 was noted. Enzyme solution containing hydrogen peroxide-free phosphate buffer was used as a control.

For the peroxidase (POD) activity, assay mixture consists of 0.1 M phosphate buffer, 0.1 mM pyrogallol, and 5 mM H_2_O_2_ with enzyme extracts (100 μl). The assay mixture was incubated for 5 min at 25°C. The reaction was stopped by adding 1.0 ml of 2.5 N H_2_SO_4_. The absorbance of indigo color formed was read at 420 nm against blank containing water in place of enzyme extract.

### Test of Colonization

Soil particles adhering to the root surface were gently removed and washed with Milli-Q water to remove any attached soil particles. The roots were cut into 1 cm long segments and 1 g of root segments was dipped into 5 ml of sterilized PBS buffer and vortex 5–6 times to release the bacteria into the buffer. Dilutions of bacterial suspensions were poured on Nutrient agar to evaluate the population of indigenous bacteria. The colony forming units (CFU) were counted after 24–48 h of incubation at 28°C±1. For colonization study, root segments (1–2cm) were stained in a solution of 0.1% acridine orange for 2–3 min followed by three succcessive washing with sterile distilled water. The stained root was placed on a glass slide with a coverslip on top of it and viewed under an epifluorescence microscope (Olympus-CKX41, Olympus, Japan) at intensity between 450 and 490 nm using 100 X objective lens and 10 X eyepiece lens. For confirming identity of colonized bacterium, ERIC-PCR was performed following the standard method [42].

### Statistical Analysis

All the experiments were conducted in triplicates and results expressed as mean ± standard deviation (SD). Data was analyzed by analysis of variance (ANOVA) followed by Duncan’s multiple range test at p = 0.05.

## Results and Discussion

### Characterization of strain

**Fig 1:**
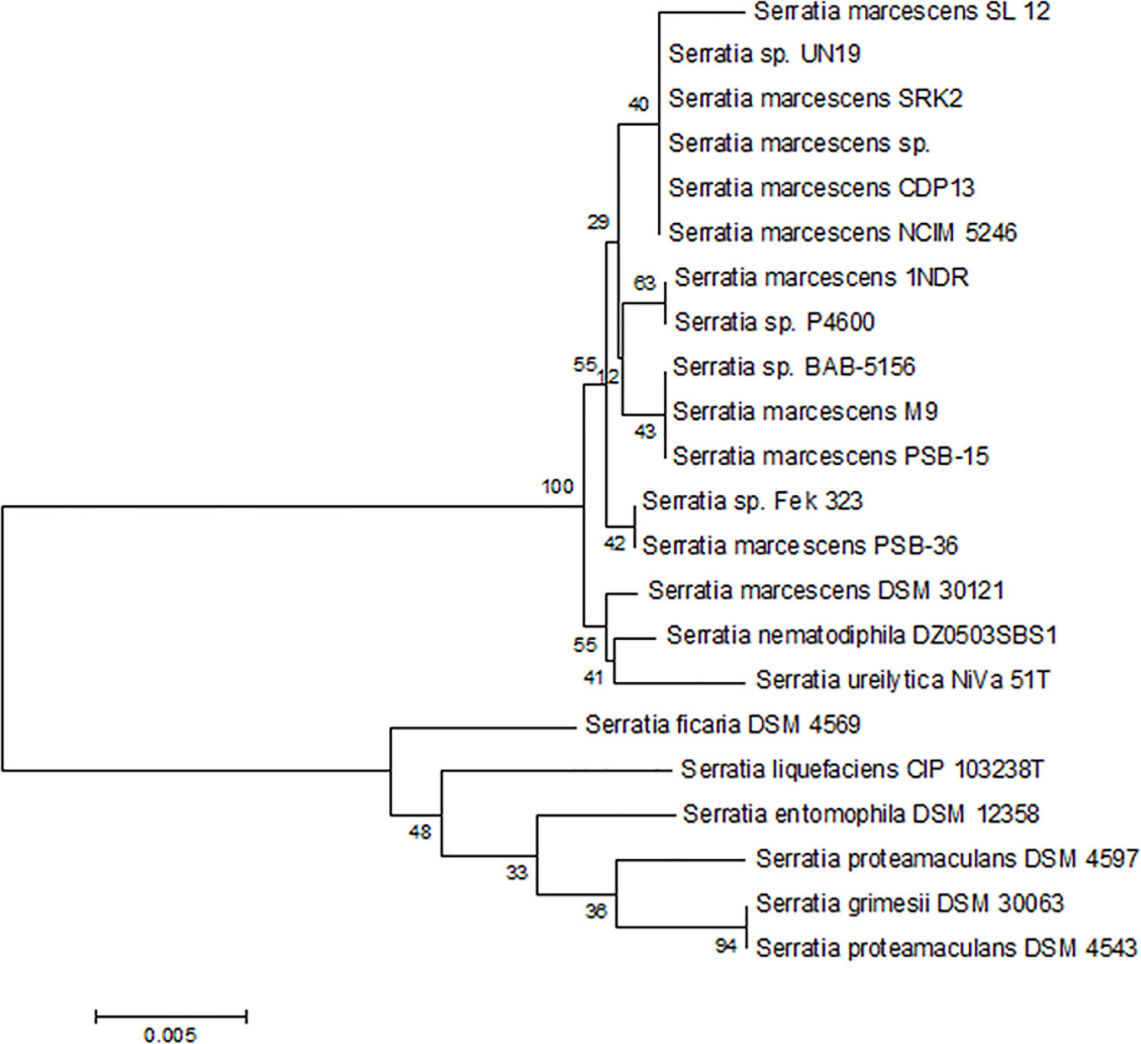
Phylogenetic tree showing the relationship of *Serratia marcescens* CDP-13 with other closely related bacterial strains and species. The 16S rRNA gene sequence of closely related species was obtained from NCBI Genbank database. The tree was constructed using neighbor- joining method of software package Mega version 6.0, at the bootstrap value of n = 500.

Based on the difference in morphology, overall ten bacterial colonies were observed on DF-agar medium supplemented with ACC as a nitrogen source and subcultured several times on selective medium (DF with ACC). Finally, luxuriantly growing bacterial isolate CDP-13 was selected for further characterization and experimental studies. The test organism was identified as *Serratia marcescens* by partial sequencing of 16S rRNA gene sequence. It showed 100% sequence similarity with *Serratia marcescens* strain SRK2 and *Serratia marcescens* strain NCIM 5246. The sequence of CDP-13 has been submitted to the Genbank under the accession number KJ950714. Phylogenetic analysis of strain CDP-13 also revealed its relatedness with several other strains of *Serratia sp*. (Fig 1).

Based on the quantitative assay, the ACC deaminase activity in CDP-13 was 172.50±7.90 nmol of α-KB/mg pr^.^/hr (Table 1). The test isolate showed more than enough ACCD activity to ameliorate salt stress which is supported by the fact that the isolate having >20 nmol of α-KB mg^-1^ h^-1^ can influence plant growth promotion due to the reduction of stress ethylene produced in plants under stress conditions [12]. Production of ACC deaminase by PGPR has an additional benefit as it reduces the level of ‘stress ethylene’ produced under stress conditions in host plants, and stimulates plant growth [43]. The breakdown products of ACC namely α- ketobutyrate and ammonia serve as a source of nitrogen and energy respectively for the associated bacteria [44]. The ACCD activity of 276 nmol α-KB mg^-1^ h^-1^ and 12 nmol α-KB mg^-1^ h^-1^ has been reported in *Serratia proteamaculans* and *S*. *quinivirans* SUD165 respectively in earlier findings [45, 46]. The growth improvement of coconut palm following inoculation of halotolerant bacterium *S*. *marcescens* KiSII showing ACCD activity was observed [47].

**Table 1:** Plant growth promoting traits of strain *Serratia marcecens* CDP-13.

Plant growth promoting traits	Activity
ACCD activity (nmol of α-KB mg^-1^Pr^.^ h^-1^)	172.50±7.90
IAA production (μg ml^-1^)	0.34±0.02
Phosphate solubilization (μg ml^-1^)	13.92±0.12
Gibberellic acid production	-
Siderophore index	+
Chitinase activity	+
HCN production	+
Ammonia production	+

ACCD, ACC deaminase; IAA, Indole acetic acid; HCN, Hydrogen cyanide

**Fig 2:**
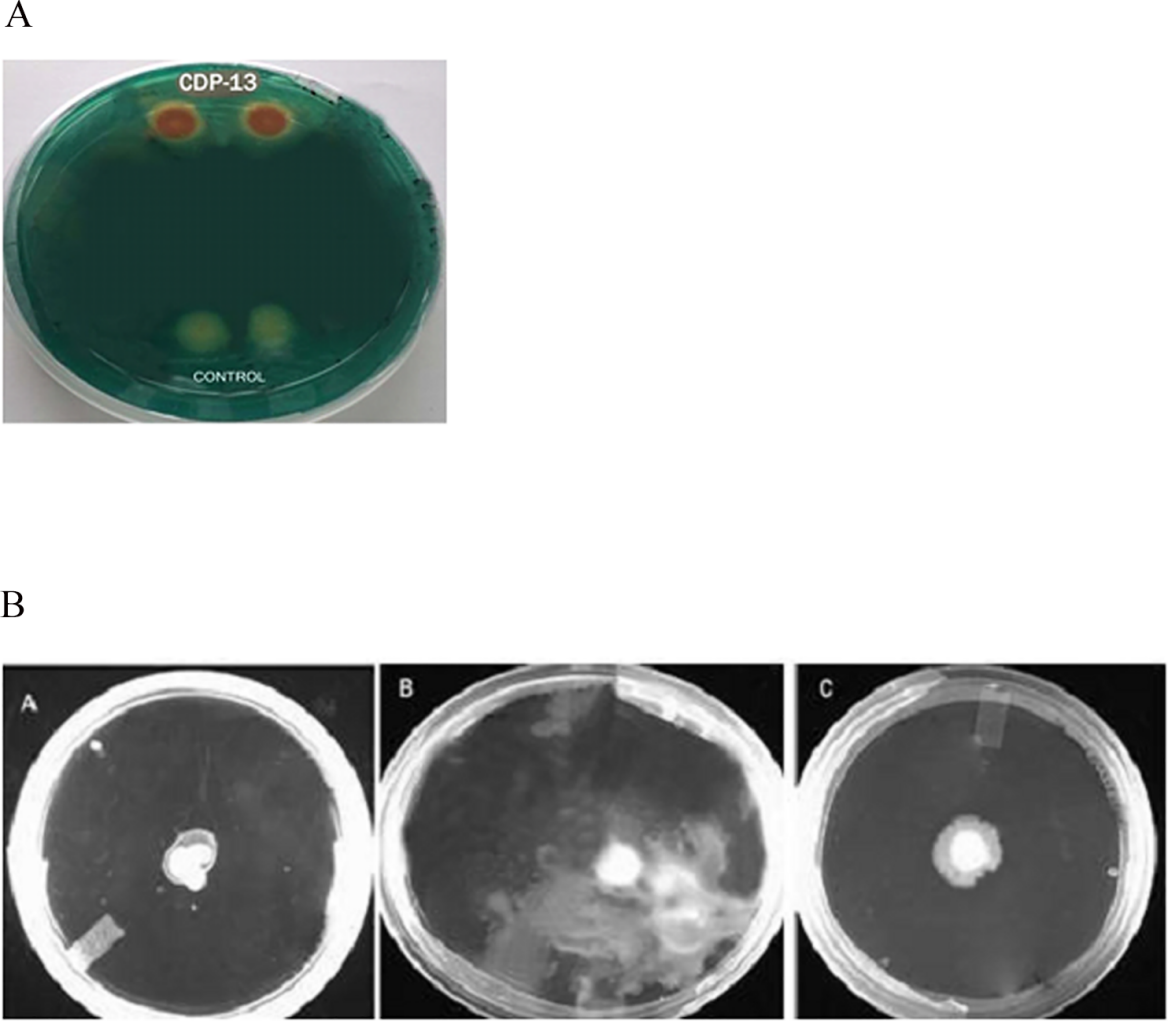
**Ability of the isolate CDP-13 to produce** (A) siderophore on CAS-agar plate and (B) to exhibit different types of motility using agar based method. Panel A, B, and C of Fig 2B represent swimming, swarming, and twitching motility respectively.

In addition to ACCD activity, CDP-13 also exhibited other PGP properties such as IAA production, phosphate solubilization, and ammonia production. The luscious growth on JNFb^-^ semi-solid agar medium was considered as positive for the test of nitrogen fixation. The appearance of clear zone on NBRIP agar medium indicated the ability of the bacterium to solubilize the inorganic phosphate. It is reported that Indian soils are normally deficient in available phosphorus, and therefore, the use of phosphate solubilizing bacteria will be more advantageous for mobilization of phosphorus efficiently in plants [48]. These results suggest that the CDP-13 can be beneficial in improving growth of wheat and other plants by providing nitrogen and phosphorous nutrition. The CDP-13 produced 0.34±0.02 μg/ml IAA, which is a valuable trait of PGPR, as this phytohormone enables the plant to develop highly organized root system by which uptake of nutrients become more efficient [49]. However, it showed negative results for another phytohormone, gibberellic acid. The test organism CDP-13 was observed to be positive for siderophore production (Fig 2A). Production of siderophore is an important trait of PGPR that influences plant growth through the suppression of fungal pathogens by rendering iron unavailable in the rhizosphere. It also facilitates iron nutrition which is also a cofactor for nitrogenase enzyme required for fixation of atmospheric nitrogen [50]. Moreover, CDP-13 showed positive results for HCN production by turning the color of Whatman filter paper no. 1 (soaked in 2% sodium carbonate in 0.5% picric acid solution) to dark brown (Table 1). The presence of chitinase activity in CDP-13 points to the potential antifungal activity. An ability of the isolate to produce siderophore, HCN, and chitinase indicates potential of the bacterium to inhibit the growth of pathogenic microorganisms (Table 1), and thus enhances plant growth indirectly by restricting the growth of pathogens.

Primary characterization of *S*. *marcescens* CDP-13 was done by different microbiological and biochemical tests. The test isolate was found to be gram (–) ve, which showed positive tests for indole, Voges-Proskauer (VP), amylase, lipase, catalase, nitrate reductase, and negative for methyl red (MR), urease, and protease production (Table 2). For antibiotic sensitivity test, it was found to be sensitive to chloramphenicol, tetracycline, gentamycin and resistant to vancomycin, kanamycin. For the test of motility, the isolate showed all three different types of bacterial motility namely swimming, swarming and twitching, which are required for chemotaxis and colonization on host surface (Fig 2B). The isolate was found to grow up to 6% NaCl supplemented medium and temperature of 45°C. Based on carbohydrate utilization ability, isolate CDP-13 was found to utilize lactose, xylose, maltose, fructose, dextrose, galactose, trehalose, melibiose, sucrose, mannose, glycerol, salicin, sorbitol, mannitol, adonitol, arabitol, rhamnose, melezitose, cellobiose, ONPG, esculin hydrolysis, inulin, citrate, and malonate utilization. Carbohydrate utilization efficacy of isolate has been summarized in Table 2. Isolate was tested for various plant polymers hydrolyzing ability and it was found to efficiently hydrolyze the β-glucosidase, cellobiohydrolase and negative for endoglucanase and pectinase. The ability to utilize the carbon sources tallied with previous reported standard strains of *Serratia marcescens* SE1 and *S*. *marcescens* MO-1 [51, 52].

**Table 2:** Biochemical and physiological properties of CDP-13 and its carbohydrate utilization pattern.

Characteristic (s)	CDP-13	Carbohydrate	Utilization
Gram reaction	-	Sodium gluconate	-
Catalase	+	Glycerol	+
Indole	+	Salicin	+
Methyl red	-	Dulcitol	-
Voges proskauer	+	Inositol	-
Amylase	+	Sorbitol	+
Lipase	+	Mannitol	+
Urease	-	Adonitol	+
Nitrate reductase	+	Arabitol	+
Temp. tolerance (°C)	45	Erythritol	-
Salt tolerance (%)	6%	α-Methyl-D-glucoside	-
Swimming	+	L-Arabinose	-
Swarming	+	Rhamnose	+
Twitching	+	Cellobiose	+
**Carbohydrate**	**Utilization**	Melezitose	+
Lactose	**+**	α-Methyl-D-mannoside	-
Xylose	**+**	Xylitol	-
Maltose	**+**	ONPG	+
Fructose	**+**	Esculin hydrolysis	+
Dextrose	**+**	D-Arabinose	-
Galactose	**+**	Citrate utilization	+
Raffinose	**-**	Malonate utilization	+
Trehalose	**+**	Sorbose	-
Melibiose	**+**	Inulin	+
Sucrose	**+**	Mannose	+

### Assessment of induced systemic resistance (ISR)

**Fig 3:**
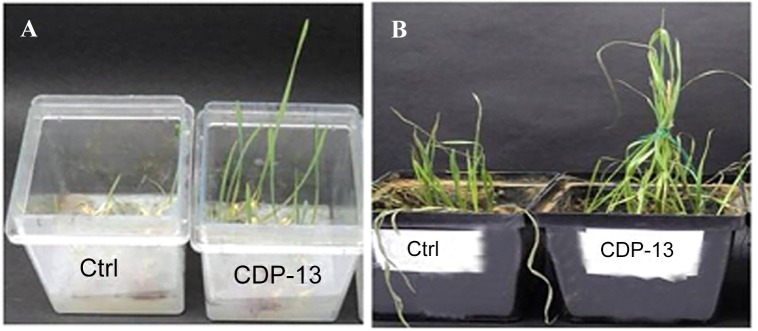
The growth of wheat plants treated with *Serratia marcesens* CDP-13 and a plant pathogen (*F*. *graminearum*). (A) The growth of bacterium and fungal treated plants on water agar medium, (B) Pot experiment showing growth of bacterium and fungus-treated plants in the soil.

Antagonistic activity of the isolate CDP-13 was tested by the antifungal assay. The test isolate inhibited growth of *Fusarium oxysporum* and *F*. *graminearum* (S1 Fig). PGPR can also protect host plant from biotic stressors by conferring 'induced systemic resistance (ISR)' which refers to the induction of defence enzymes and proteins in host plants, which in turn decreases disease severity caused by infecting pathogens. Therefore, *S*. *marcesens* CDP-13 was tested for elicitation of ISR in wheat plants. Inoculation of the wheat plants with test isolate showed significant (p = <0.05) reduction of disease symptoms in *F*. *graminearum*-treated plants as compared to uninoculated control plants. For the above test, plants were grown aseptically in water agar (Fig 3A). The severity of disease caused by fungal infection was visually determined on a scale of 0–5. The plants inoculated with the isolate CDP-13 exhibited the lowest number of symptomatic effects with the pathogen inoculation as compared to the untreated control (S1B Fig). Development of ISR by CDP-13 was also tested by conducting pot studies which showed similar results to that of the water-agar test described above (S1C Fig). The isolate was found to be effective for fungal resistance in the soil used in pot experiment (Fig 3B). The previous study has demonstrated that plant growth promoting rhizobacteria *Serratia marcescens* 90–166 mediates the induced systemic resistance (ISR) to fusarium wilt disease of cucumber (*Cucumis sativus* L.) caused by *Fusarium oxysporum* [53]. To date, only a few study has examined the role of PGPR to induce systemic resistance against the disease of their host plants. However, the detailed mechanism of disease resistance in plants is still lacking. Future studies should be designed to explore the tissue specific induction of systemic resistance related to the reduction of fungal infection by CDP-13. Moreover, tissue-specific expression of pathogenesis-related (PR) protein will shed light on the mechanism involved in the generation of resistance against pathogen following inoculation of *S*. *marcescens* CDP-13.

### Effect of *S*. *marcescens* CDP-13 on plant growth under salt stress conditions

**Fig 4:**
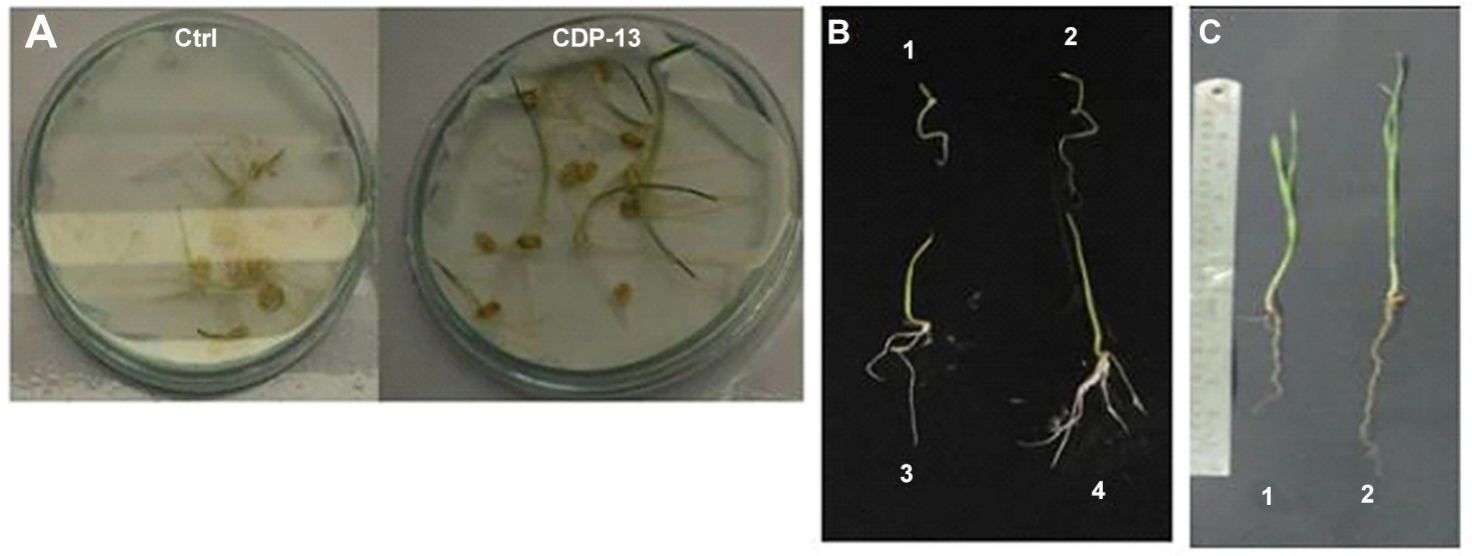
The growth of bacterium-inoculated plants was compared at different time intervals with their control-counterparts. (A) plate based screening, (B) sprouted wheat seedlings 1,2 control; 3,4 CDP-13 inoculated seedlings, (C) growth after 15^th^ days of germination; Panel 1 is uninoculated control plants, whereas panel 2 shows CDP-13 inoculated plant.

**Fig 5:**
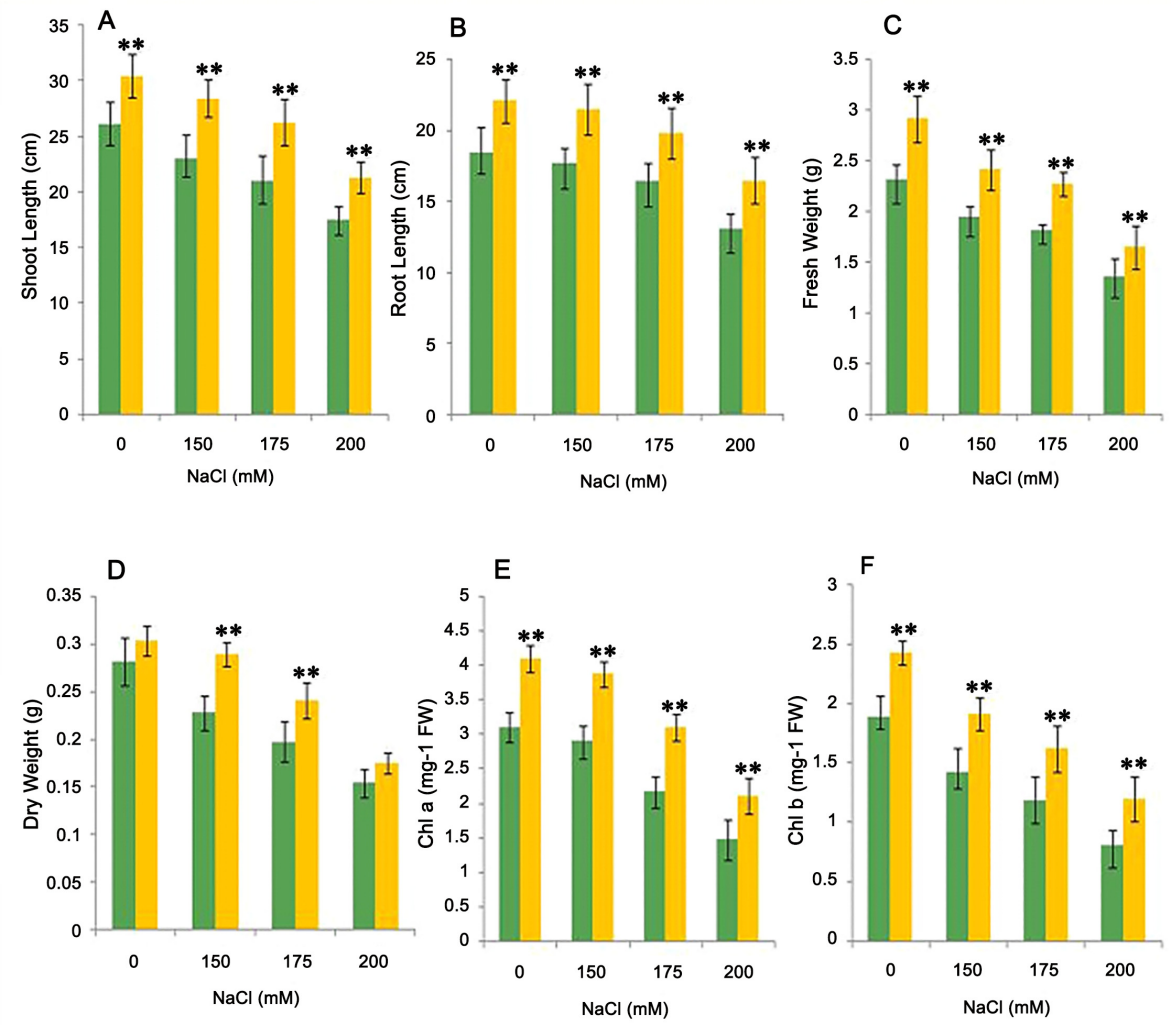
Effect of inoculation with isolate CDP-13 on plant biomass and chlorophyll content under different concentrations of NaCl. Different plant growth parameters were tested which included (A) Shoot length. (B) Root length (C) Fresh weight (D) Dry weight (E) Chlorophyll a (F) Chlorophyll b. Values are mean ± SD of triplicate sets (n = 15). *Double asterisk* ** represent the significant difference according to Duncan multiple range test (*p = 0*.*05*) as compared to control.

The production of ACC deaminase modulates the ethylene level in plants which contributes to the stress tolerance in plants [54, 55]. Therefore, the test isolate was evaluated for its ability to ameliorate salt stress in the wheat plant. Inoculation with the isolate CDP-13 enhanced the growth of wheat plants treated with different levels of salt (NaCl) stressors. The growth was assessed by measuring the various physiological parameters of the wheat plant and photosynthetic pigments. (Fig 4A–4C) demonstrates the growth of bacterium-inoculated plants at various time intervals. Inoculation with bacterium CDP-13 significantly enhanced the wheat growth (approx. two to three folds) as compared to control plants. Furthermore, the growth of plants inoculated with CDP-13 was evaluated under salt stress by setting up pot experiments. Physiochemical properties of the soil used for the pot study are summarized in Table 3. In pot studies, bacterium inoculation resulted in 17–49% increase in all the growth parameters tested (Fig 5A–5F). However, the maximum growth varied for different parameters at different concentration of salt tested in this study. Shoot length, fresh weight and chlorophyll a content were highest (25, 25.41, and 43.5% respectively) at 175 mM, whereas root length and chlorophyll b content showed the highest increase (26 and 48.5% respectively) at 200 mM NaCl. Data on dry weight indicate that inoculation with the isolate resulted in significant (p = 0.05) increase in dry weight of 26.75% and 22.33% at 150 and 175 mM NaCl stress as compared to respective control plants (Fig 5D).

**Table 3:** Physiochemical properties of soil used for pot study.

Parameter	Value
pH	7.20±0.02
Electrical Conductivity (EC)	0.158±0.01ds m^-1^
Olsen P	32.8 ±1.4mg kg^-1^
Total N	63 ±2.1 mg kg^-1^
Total K	126.7 ±4.2 mg kg^-1^
Zn	0.213 ±0.001mg kg^-1^
Cu	0.131 ±0.001mg kg^-1^
Fe	2.76 ±0.03 mg kg^-1^
Mn	0.963 ±0.02 mg kg^-1^

The increase in enhanced chlorophyll content in a bacterium-treated plant under salt stressors signifies that bacterial treatment counteracts the effect of salinity stress on photosynthetic activity and growth of the plant. Improvement in photosynthetic activity by PGPR has been reported in some of the earlier studies [56, 57]. The observed increase in plant growth by ACC deaminase-producing *Serratia marcecens* CDP-13 is also supported by other observations, where ACC deaminase-producing bacteria were shown to enhance plant growth by lowering the ethylene level [58, 59].

### Effect of test isolate on oxidative damage under salt stress

**Fig 6:**
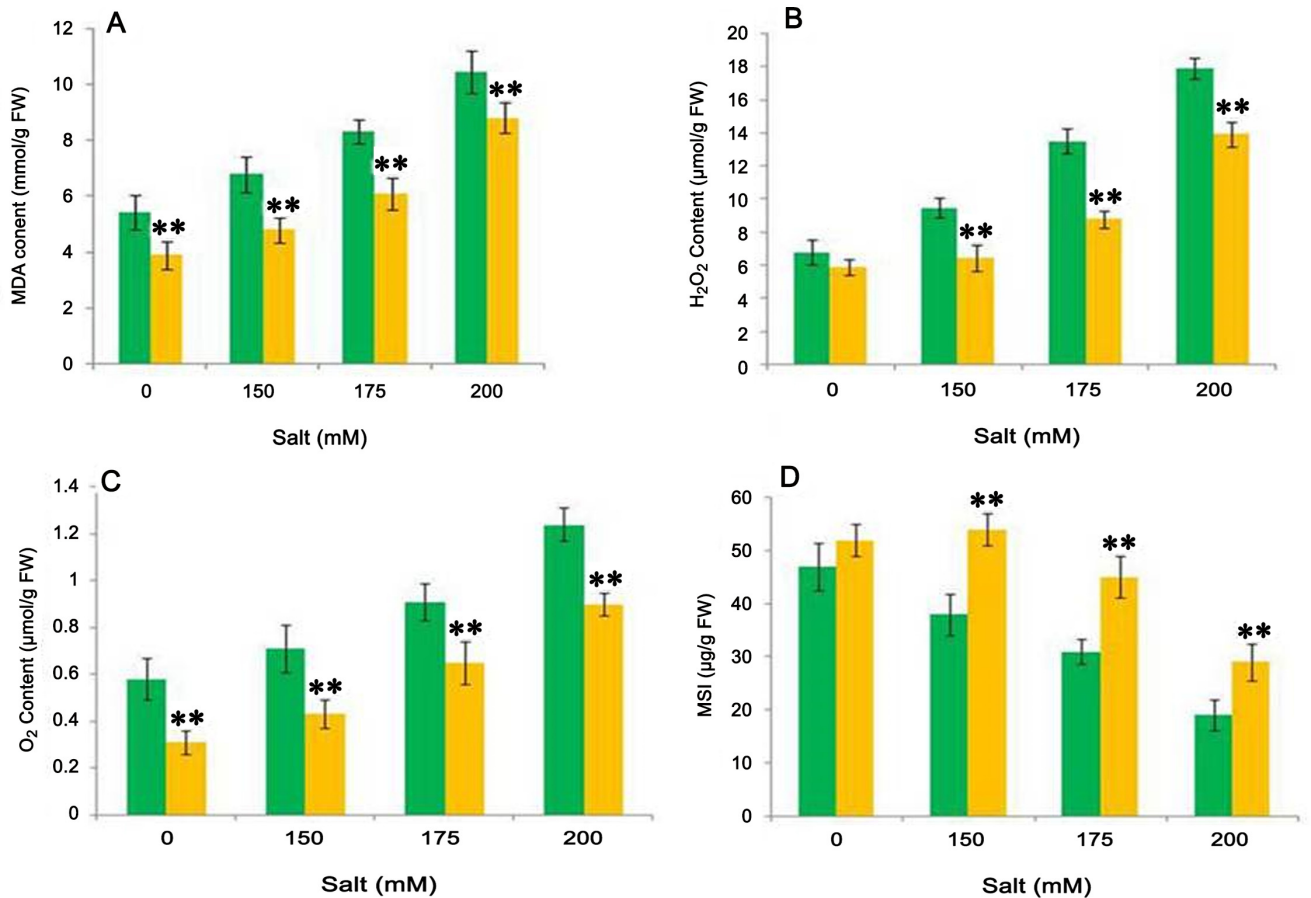
**Effect of inoculation with isolate CDP-13 on salinity induced oxidative damages in wheat plants with respect to generation of** (A) MDA content (B) H_2_O_2_ content (C) O_2_^-^, and measurement of (D) Membrane stability index. Values are mean ± SD of triplicate sets (n = 15). *Double asterisk* ** represent the significant difference according to Duncan multiple range test (*p = 0*.*05*) as compared to control counterpart.

As a consequence of increasing salt stress, increased oxidative damage to lipid results in increased malondialdehyde (MDA) content, which causes an increase in membrane permeability, exosmosis of electrolytes and finally higher cell injuries [60]. Therefore, we estimated the level of MDA content in response to salt stress in presence and absence of test organism. The amount of MDA content increased (25% to 92%) with an increase in salt concentration in uninoculated plants. However, bacterium inoculation significantly decreased (p = 0.05) the MDA content in NaCl-treated plants. The highest decrease (p = 0.05) with 41% of total MDA content was observed at 150 mM NaCl stress against the uninoculated control plants (Fig 6A). Thus, the result of the present study showed that inoculation with isolate CDP-13 lowers cell injuries and increases tolerance of host plants to salt stress.

In addition, oxidative damage was assessed by measuring the proportion of H_2_O_2,_ superoxide anion (O_2_^-^) and membrane stability index (MSI). Plants treated with NaCl showed higher accumulation of H_2_O_2_ as compared to bacterium -inoculated plants. With an increase in salinity level from 0 to 200 mM, the content of H_2_O_2_ increased by 39 to 62%. However, bacterium inoculation significantly (p = 0.05) reduced the H_2_O_2_ content in the range of 10–57% (Fig 6B). Similarly, we observed an increase in superoxide anion (O_2_^-^) in the range of 22–53%, whereas bacterium inoculation decreased the O_2_^-^ accumulation by 40–80% as compared to respective control plants (Fig 6C). Bacterium-inoculated plants showed higher membrane stability between 11 and 42% as compared to control plants treated with NaCl stress (Fig 6D). Our data strongly suggest that salt stress can induce membrane lipid peroxidation resulting in loss of membrane stability, and caused the higher electrolytic leakage. However, bacterium CDP-13-treated plants exhibited relatively less membrane injury and lipid peroxidation levels compared to the untreated control plants. These findings reveal that ROS scavenging became efficient following the bacterial application for enhancing salinity tolerance in wheat. Our results are in agreement with the previous study which demonstrated that beneficial plant growth promoting rhizobacteria protect the rice seedlings against the salinity induced oxidative damages [61].

### Test for the production of antioxidant enzymes in plants

**Fig 7:**
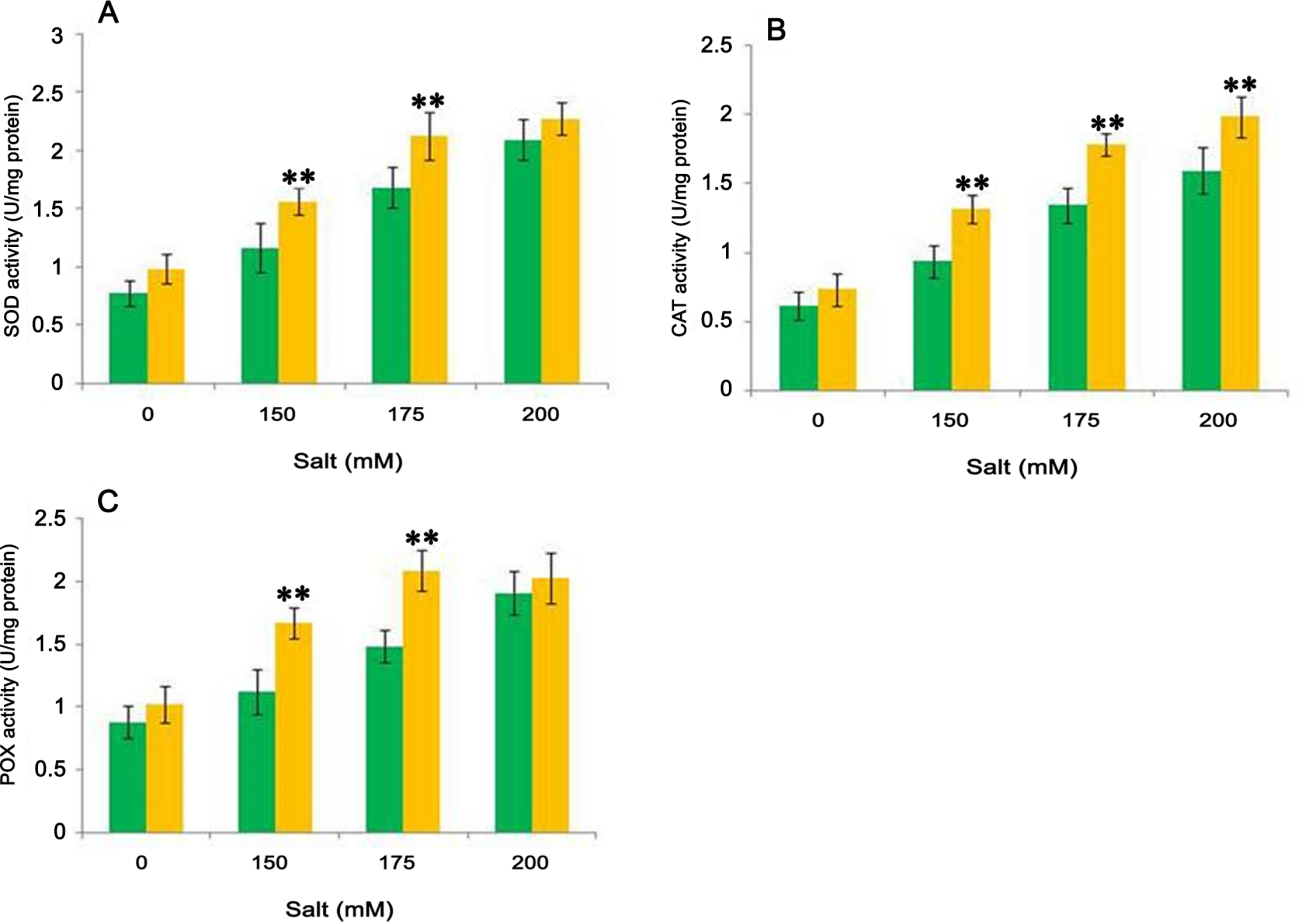
Effect of inoculation with isolate CDP-13 on antioxidative enzymatic activities in bacterium treated and control plants under tested salt stress conditions. (A) SOD activity (B) CAT activity(C) POX activity. Values are mean ± SD of triplicate sets (n = 15). *Double asterisk* ** represent the significant difference according to Duncan multiple range test (*p = 0*.*05*) as compared to control.

A significant increase in the anti-oxidative enzyme was observed following inoculation of the isolate CDP-13. As shown in Fig 7, bacterium inoculation significantly enhanced the SOD activity as compared to respective control plants. The level of SOD increased by 34% in salt-treated (150 mM) plants inoculated with the bacterium CDP-13 as compared to that of respective control plants (Fig 7A). Alleviation of oxidative damage by scavenging ROS with the use of antioxidant enzymes is an important strategy of plants for increasing stress tolerance. Increased activities of SOD in bacterium-treated plants can play a crucial role in scavenging superoxide radicals during salinity stress. Our results show that anti-oxidative activity is well protected in bacterium-treated plants unlike that of control plants.

In addition to SOD, the level of other antioxidant enzymes also increased in plants treated with the test organism at different concentration of NaCl. Increase in salinity, significantly increased the CAT activity in uninoculated plants, while it was higher in the bacterium-inoculated plants. In the presence of bacterial inoculation, CAT activity significantly (p = 0.05) increased by 19.6, 40.8, 33, 24.5% at 0, 150, 175 and 200 mM NaCl stress compared to respective control plants (Fig 7B). The increase in CAT activity in inoculated plants was probably due to the fact that the bacterial inoculation stimulated the synthesis of the anti-oxidative enzyme. Increase in salinity from 150 to 200 mM NaCl, enhanced the POX activity (27 to 80%) in un-inoculated plants, as compared to non-salt treated ones. The POX activity increased in the range of 63 to 110% in bacterium-inoculated plants as compared to un-inoculated plants at 0 mM NaCl. Similar to the activity of CAT enzyme, the activity of POX also increased significantly (p = 0.05) up to 49% in bacterium-treated plants against uninoculated plants under salt stress (Fig 7C). In *Triticum aestivum* L., salt stress resulted in the increase of antioxidant enzymes CAT and POX, but activities of these enzymes were significantly higher in the bacterium-treated plants. It has been shown in earlier study that the bacterial inoculation leads to increase in catalase activity and accumulation of proline (discussed in following paragraph) improves salt tolerancein olive tree leaves [62]. The increase in POX activity also significantly contributes to the plant development as the POX enzyme is involved in lignin formation during plant growth and defense responses [63]. The results of antioxidant enzyme assays in present study suggested that the inoculation of PGPR mitigate the salt-induced damages in plants through enhanced activities of antioxidant enzymes. Our results are corroborated by earlier reports which illustrated that increase in antioxidant activities helps plants to cope with salinity like abiotic stress [64, 65].

### Biochemical analysis of plants treated with CDP-13 under salt stress condition

**Fig 8:**
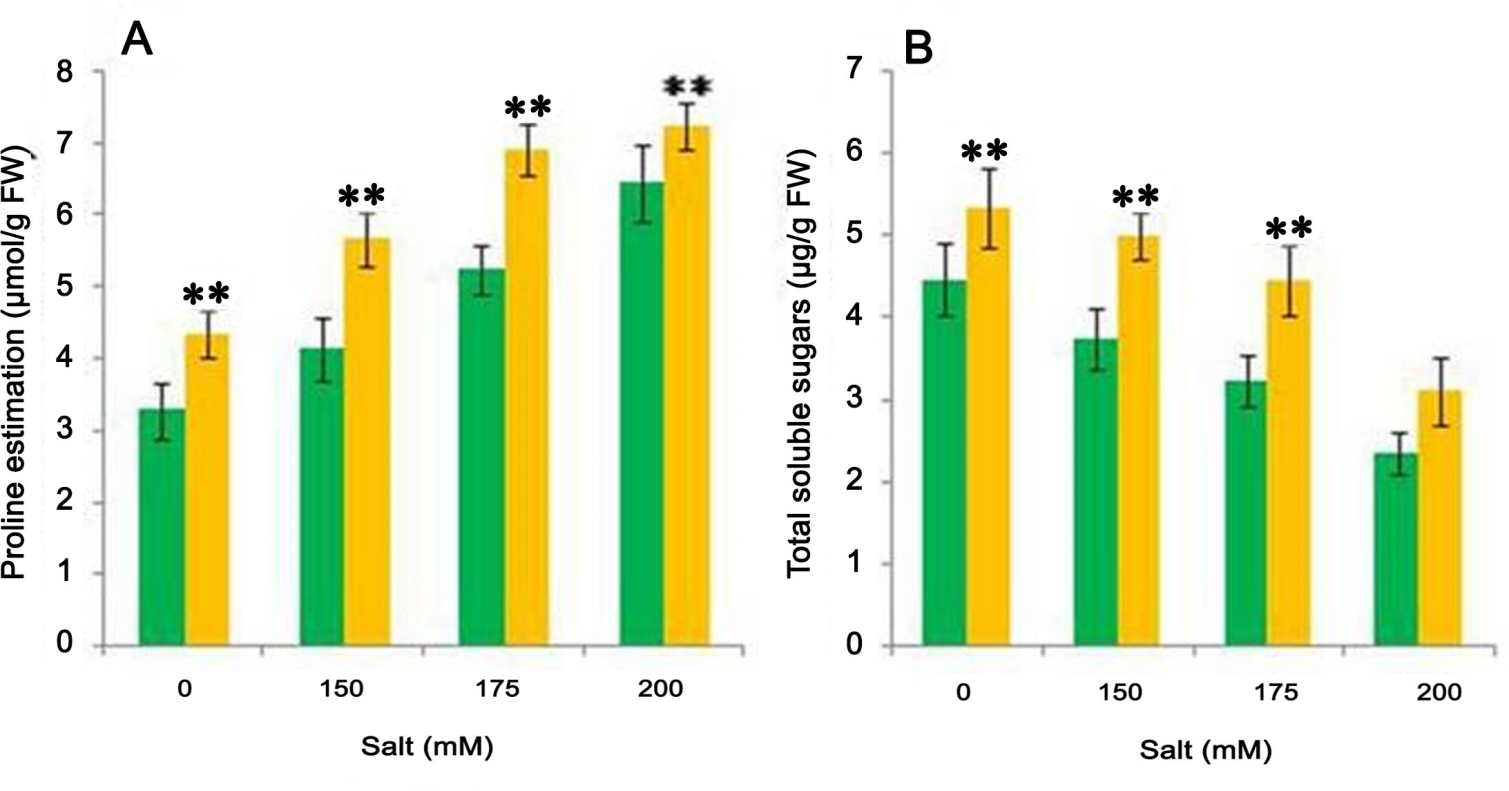
Effect of bacterium inoculation on the production of various osmolytes in plants supplemented with various concentration of NaCl. (A) Proline (B) Total soluble sugar under different concentration of NaCl (0 mM, 150 mM, 175 mM, 200 mM). Values are mean of three replicates ± SD (n = 15). Significant difference according to Duncan multiple range test (*p = 0*.*05*) compared to control has been denoted by *Double asterisk* **.

Maintaining the osmotic adjustment is essential for alleviating the effect of salt stress on plant growth and yield. Osmotolerance induced by accumulation of compatible solutes ‘osmolytes’ protect the plant cells against salinity-induced desiccation and cell injury. The accumulation of certain osmolytes under salt stress condition was evaluated in bacterium-treated plants. Inoculation of the isolate CDP-13 significantly enhanced the contents of organic solutes (osmotica) in salt-stressed wheat plants, which can improve the tolerance to salinity in plants. We observed significant increase (32 to 37%, p = 0.05) in proline content in CDP-13-treated plants at different concentration of NaCl, being highest (37.13%) at 150 mM (Fig 8A). Proline has been reported to reduce the enzyme denaturation caused by NaCl and other stress. The accumulation of proline alleviates the salt stress-induced reduction in the activities of antioxidant enzymes [66]. It is considered not only as a compatible solute and osmoprotectant but also a hydroxyl radical scavenger. Under stress conditions, proline stabilizes proteins, membranes, and sub-cellular structures and protects cellular functions by scavenging ROS. Moreover, a significant change in total soluble sugar (TSS) content in wheat leaves stressed with different salt treatments was also observed in the presence of bacterial isolate. The increase in salinity from 150 mM to 200 mM decreased the soluble sugar content in the range of 20 to 90%. However, presence of bacterial isolate CDP-13 significantly (p = 0.05) increased the soluble sugar content. The highest significant (p = 0.05) accumulation of TSS (38.62%) was observed at 175 mM salt stress in bacterized plants as compared to uninoculated control plants treated with respective salt stress (Fig 8B). Accumulation of soluble sugars plays a key role in the osmoprotection, osmotic adjustment, and radical scavenging. Therefore, the increase in soluble sugar in the present study supports that they are essential to alleviating salinity stress either via osmotic adjustment or by conferring some desiccation resistance to plant cells. Increase in total soluble sugar is another defense approach to counteract the effect of salinity stress [67]. Therefore, an increase in TSS content following inoculation of *S*. *marcescens* CDP-13 significantly contribute to plant growth against salt stress through modulating the defense strategies.

**Fig 9:**
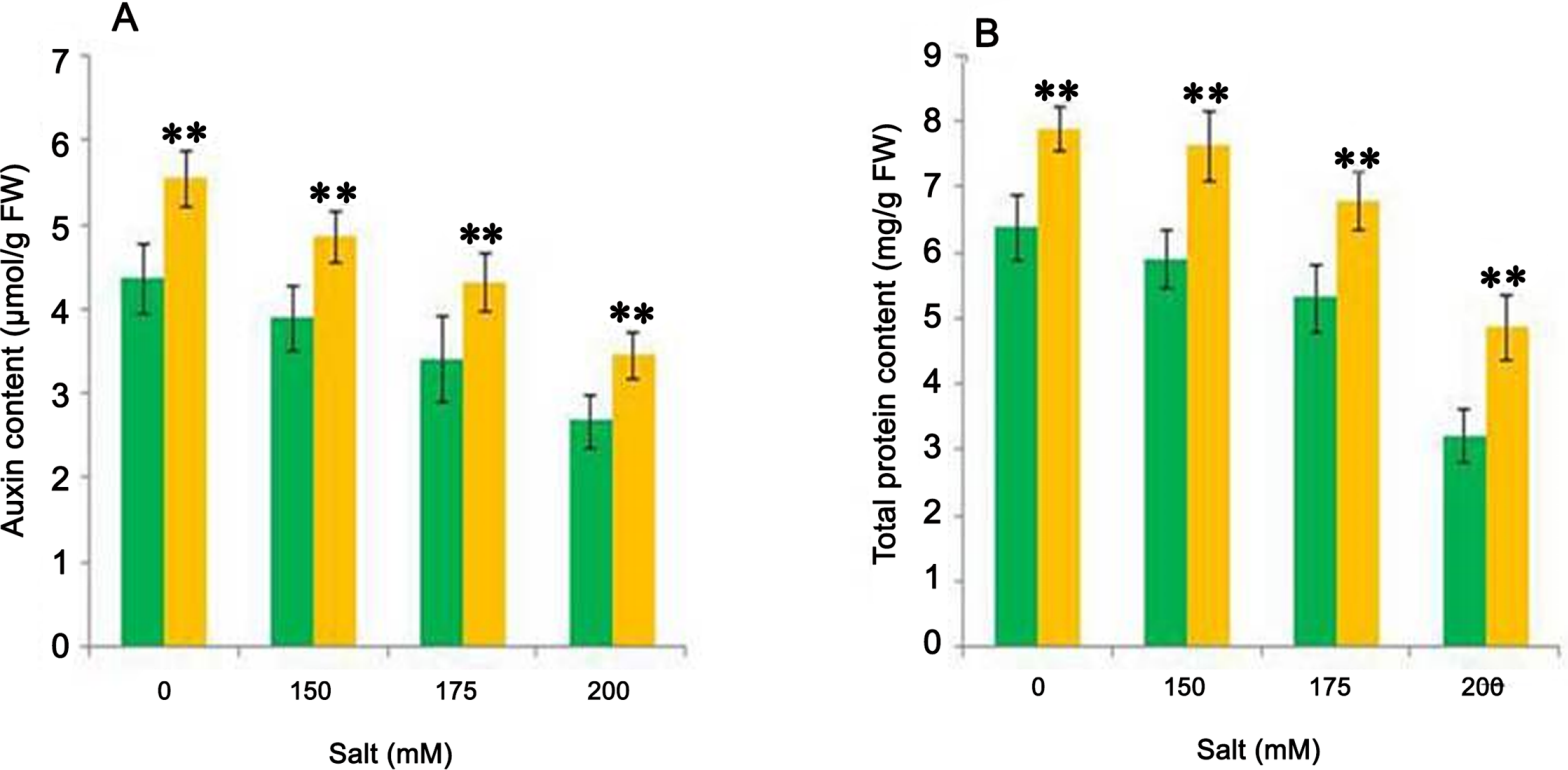
**Effect of bacterial inoculation on the production of** (A) Auxin and (B) Total protein content under different NaCl concentration (0 mM, 150 mM, 175 mM, 200 mM). Values are mean of three replicates ± SD (n = 15). Significant difference according to Duncan multiple range test (*p = 0*.*05*) compared to control has been denoted by *Double asterisk* **.

Other properties useful in the protection of plants from stress condition are the maintenance of auxin concentration and total protein content which are severely affected by the stressors. As shown in Fig 9A and 9B, the test organism significantly increased (p = 0.05) the auxin content in wheat plants by 25 to 29%, and by 23 to 52% in total protein content as compared to respective uninoculated control plants. The decrease in IAA level in plants under stress conditions resulted in significant reduction in germination, plant growth and developments [68]. Auxin plays a major role in regulating the several aspects of plant growth and development. Plants subjected to salt stress produce excess amount of this hormone as an adaptive mechanism to counteract the stress situation [69]. However, along with normal metabolic activities, this excess production of auxin requires extra metabolic energy. This extra energy consumption for production of auxins resulted in decreased plant growth. Therefore, auxin produced by PGPR can minimized the energy requirement of plant and act as effective biocatalyst to enhance the plant growth under salt stress. It is reported that exogenous application of auxin improves the plant growth through vascular tissue development, cell elongation, organogenesis etc. [70]. The ability of the isolate CDP-13 to produce auxin might be responsible for growth and salinity tolerance of wheat plants.

### Colonization of *Serratia marcescens* CDP-13 in wheat plants

**Fig 10:**
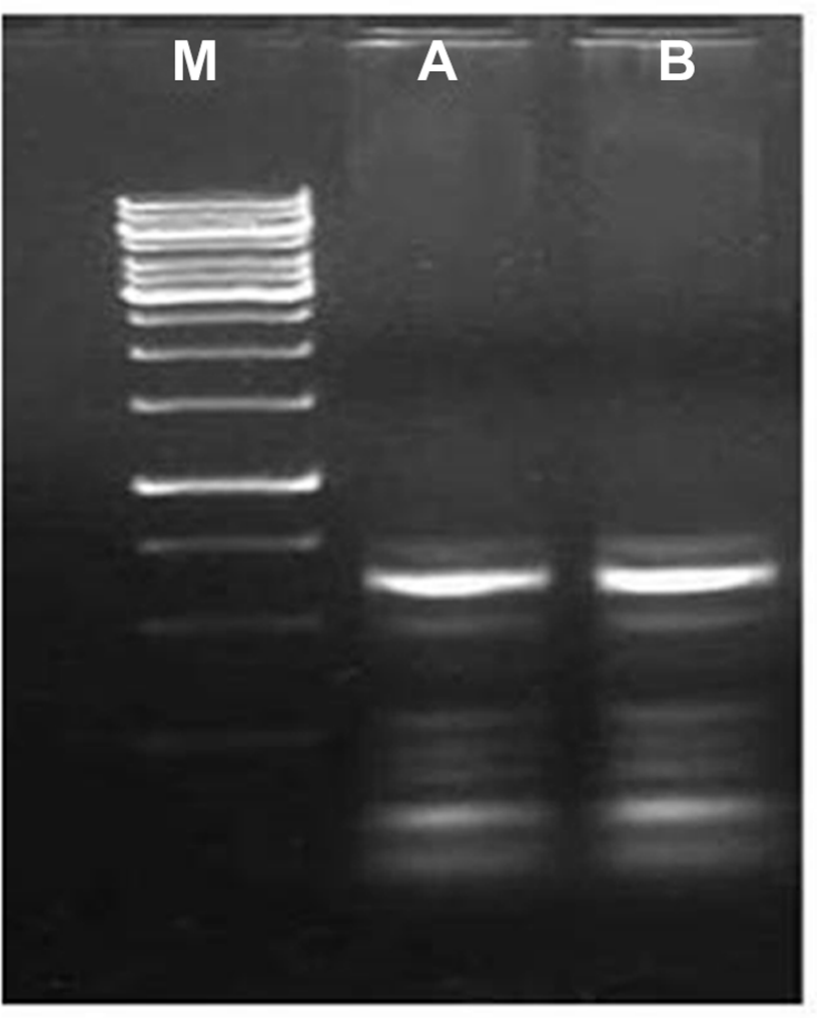
Assessment of the efficiency of colonization of isolate CDP-13 based on ERIC-PCR profile of bacterium colonized in wheat plants. (Lane M: DNA ladder SM0311, Lane A: control DNA, Lane B: CDP-13 DNA of *S*. *marcecens* isolated from inoculated plant).

The optimal outcome of plant-PGPR interaction in terms of increased growth and yield of the plant depends on the extent of successful colonization of associating bacteria. Therefore, we evaluated the efficiency of *S*. *marcescens* CDP-13 to colonize wheat plant employing different approaches. An ability of colonization was tested after 15 days of inoculation of wheat plantlets with the test isolate. Isolation of colonized bacterium after 15 days of inoculation detected associative bacteria in the range of 1.1×10^4^ CFU/g of the root. However, no bacterial colonies were recovered from uninoculated control plants. Secondly, the bacteria on plant root surface were visualized by staining with acridine orange through fluorescence microscopy. No bacterial cells were observed on the root surface of un-inoculated plants, whereas roots of inoculated plants showed a large number of fluorescent cells on staining with acridine orange (Figure not shown). Visualization of bacterial cells in inoculated plant roots using fluorescent dye acridine orange has been used to track the microbial colonization [71]. Further, the isolated bacterium from inoculated plants was subjected to ERIC-PCR-based fingerprinting which revealed the identical banding pattern from total genomic DNA of treated plants to that of a pure culture of bacterium CDP-13 (Fig 10). ERIC-PCR is a robust method to type and confirm the identity of bacteria based on the DNA banding pattern in agarose gel [72,73]. Thus, these results indicate the ability of CDP-13 to colonize roots of wheat plants.

## Conclusions

From the result of the present study, it can be concluded that the addition of a rhizobacterium with multifarious plant growth promoting properties act synergistically to mitigate the salt-induced damages and enhance the salinity tolerance in wheat plant. Application of *S*. *marcescens* CDP-13 also restores membrane integrity by minimizing oxidative damages and promotes plant growth under various levels of salt (NaCl) stress. In addition, it is also effective to suppress the fungal pathogens and augment the induced systemic resistance to cope with biotic stress responses. The strain CDP-13 can effectively induce the physiological and biochemical parameters in wheat plants allowing them to cope with salinity-induced toxicity and maintains fundamental metabolic capacity under salt stress. The test isolate increases the anti-oxidative defense machinery under various salinity levels, which suggests the induction of a systemic response and provides a valuable insight of microbial mediated enhanced salinity tolerance. Further studies on the potential of this isolate to enhance the plant growth at field level would strengthen the possibility of using the isolate CDP-13 as an alternative for organic fertilizers and pesticide.

## Supporting Information

S1 Fig Inhibition of fungal growth by isolate *Serratia marcecens* CDP-13. (A) Dual culture assay (B) Degree of disease severity in control and treated plants by water agar assay (C) Degree of disease severity in pot assay.
(DOCX)
