# Supplementary figures and images for: The Multifarious PGPR Serratia marcescens CDP-13 Augments Induced Systemic Resistance and Enhanced Salinity Tolerance of Wheat (Triticum aestivum L.)
Source: PLoS One. 2016 Jun 20;11(6):e0155026. doi: 10.1371/journal.pone.0155026 (PMC4913913; doi:10.1371/journal.pone.0155026)

S1Fig.


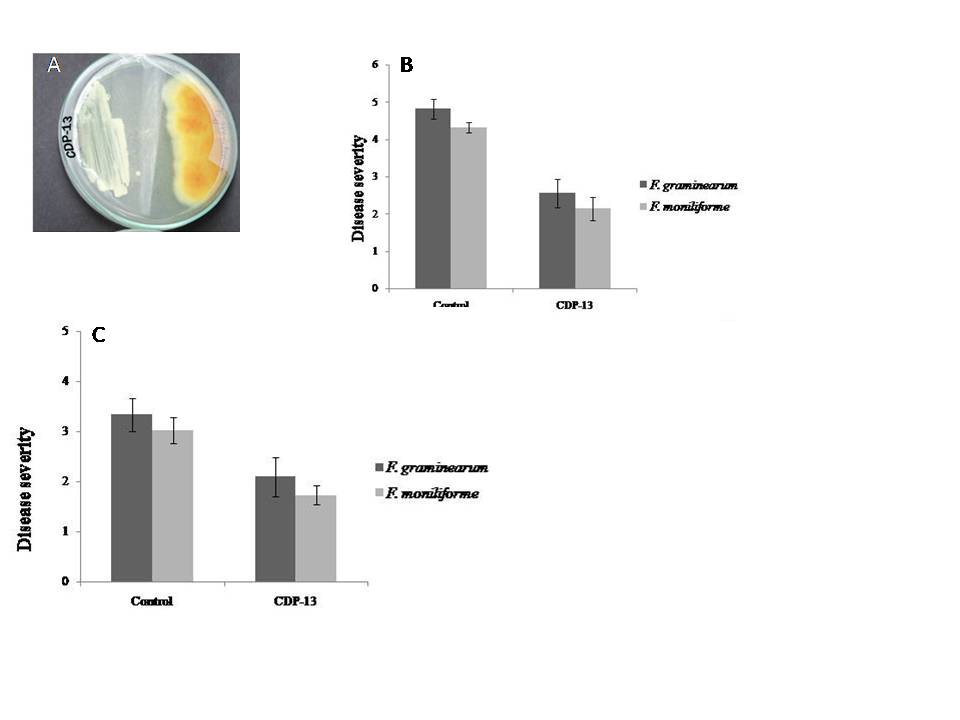

Supplement: S1 Fig — (A) Dual culture assay (B) Degree of disease severity in control and treated plants by water agar assay (C) Degree of disease severity in pot assay. (DOCX) [file pone.0155026.s001.docx]
